# A Dynamic Model of Opioid Overdose Deaths in Canada during the Co-Occurring Opioid Overdose Crisis and COVID-19 Pandemic

**DOI:** 10.3390/ijerph21040442

**Published:** 2024-04-04

**Authors:** Rifat Zahan, Nathaniel D. Osgood, Rebecca Plouffe, Heather Orpana

**Affiliations:** 1 Department of Computer Science, University of Saskatchewan, Saskatoon, SK S7N 5A2, Canada; rifat.zahan@usask.ca (R.Z.); nathaniel.osgood@usask.ca (N.D.O.); 2 Centre for Surveillance and Applied Research, Public Health Agency of Canada, Ottawa, ON K1A 0K9, Canada; rebecca.plouffe@phac-aspc.gc.ca; 3 School of Psychology, University of Ottawa, Ottawa, ON K1N 6N5, Canada

**Keywords:** opioids, overdose, fentanyl, pandemic, modelling

## Abstract

With over 40,000 opioid-related overdose deaths between January 2016 and June 2023, the opioid-overdose crisis is a significant public health concern for Canada. The opioid crisis arose from a complex system involving prescription opioid use, the use of prescription opioids not as prescribed, and non-medical opioid use. The increasing presence of fentanyl and its analogues in the illegal drugs supply has been an important driver of the crisis. In response to the overdose crisis, governments at the municipal, provincial/territorial, and federal levels have increased actions to address opioid-related harms. At the onset of the COVID-19 pandemic, concerns emerged over how the pandemic context may impact the opioid overdose crisis. Using evidence from a number of sources, we developed a dynamic mathematical model of opioid overdose death to simulate possible trajectories of overdose deaths during the COVID-19 pandemic. This model incorporates information on prescription opioid use, opioid use not as prescribed, non-medical opioid use, the level of fentanyl in the drug supply, and a measure of the proportion deaths preventable by new interventions. The simulated scenarios provided decision makers with insight into possible trajectories of the opioid crisis in Canada during the COVID-19 pandemic, highlighting the potential of the crisis to take a turn for the worse under certain assumptions, and thus, informing planning during a period when surveillance data were not yet available. This model provides a starting point for future models, and through its development, we have identified important data and evidence gaps that need to be filled in order to inform future action.

Under the guidelines of the International Committee of Medical Journal Editors (www.icmje.org, accessed on 23 March 2023) about the dissemination of information relevant to a public health crisis, a summary of these results was made available to stakeholders and the public ahead of publication through a biannual technical report, and is available at the following address: https://www.canada.ca/en/health-canada/services/opioids/data-surveillance-research/modelling.html (accessed on 23 March 2023).

## 1. Introduction

Both Canada and the United States are experiencing overdose crisis from opioids and other drugs. In 2016, Canada declared the opioid crisis a national public health concern, while British Columbia declared a public health emergency [1]. Prior to the COVID-19 pandemic, opioid, and other drug poisoning were at higher levels than ever before: between January 2016 and December 2019, there were almost 15,000 apparent opioid-related overdose deaths, growing to more than 40,000 such deaths by June 2023 [2]. At the beginning of the COVID-19 pandemic, there was concern that changes related to measures to reduce the transmission of the SARS-CoV-2 virus may further exacerbate substance-related harms, including those associated with opioids [3]. This paper describes the development of an initial dynamic model of opioid use and overdose death constructed with the goal to generate scenarios of how the opioid crisis may evolve during the concurrent COVID-19 pandemic in Canada.

The opioid crisis has been characterised as a complex health and social phenomenon that needs a collaborative, comprehensive, compassionate, and evidence-based response [1]. As a public health problem, the opioid crisis operates at multiple levels, ranging from individual behaviours, to peer interactions, to community characteristics, and to policies and programs at the local, provincial, and federal public levels. Such different levels interact with each other and impact how the opioid crisis evolves over time [4]. Moreover, this complex system had the potential to be affected by changes at all levels during the COVID-19 pandemic.

A systems science approach recognizes that complex systems arise from a large set of distinct but interconnected elements that interact with each other, that such a system exhibits an emergent behaviour distinct from the effects of single elements that evolve and adapt over time [5]. Dynamic mathematical models can be used to describe a system that evolves over time by characterising the components of a system and the relationships between them. As the opioid crisis is dynamic and complex in nature, a systems science approach can help society to better describe and understand it [6]. Although dynamic models have been widely used in epidemiological, public health, and health policy areas to model infectious or non-infectious disease outbreaks and guide public policy, their use with more complex bio-psychosocial behaviour such as opioid use and overdose deaths is more limited in comparison [7,8]. 

A systematic review of studies up to 2019 and published in 2021 identified a single model relevant to the Canadian context [7]. A literature review conducted by the investigator team prior to developing the current model in early 2020 identified relatively few dynamic models of opioid use and overdose in Canada, and no dynamic models of the opioid crisis specifically from a Canadian perspective. Models of opioid use and/or overdoses in the Canadian context have primarily focussed on specific forms of health care or health care interventions. Baia et al. used discrete-event simulation to model the resource capacity needed in the emergency department under different scenarios of changing demands, including increased demands due to substance use in general [9]. McGregor et al. applied system dynamics modelling to evaluate the potential role of diverting musculoskeletal pain clients to chiropractic care in reducing opioid use and deaths [10]. Marks et al. used data from three cohort studies, including one from Vancouver, British Columbia, to model how increasing opioid agonist therapy might reduce the annual incidence of injection drug use [11]. Irvine et al. used a Bayesian hierarchical latent Markov process model to estimate the cumulative impact of three interventions on opioid overdose deaths in British Columbia [12]. To the best of our knowledge when the present model was developed, no national-level dynamic model was available to simulate the dynamics of opioid use and overdose death in Canada in the context of the opioid overdose crisis.

When COVID-19 cases began to rise in Canada, and public health measures were put in place to reduce the rates of transmission of the virus, there was potential for the complex system of the opioid crisis in Canada to be disrupted. This could have included changes in the illegal drug supply, reduced access to harm reduction and treatment services aimed at reducing opioid related harms, such as safe consumption sites or opioid agonist therapy, and behavioural changes by individuals in response to the pandemic situation [3,13,14]. Given the novel situation, it was unclear how the COVID-19 context may impact the ongoing opioid crisis. We identified the potential for a dynamic model of opioid use and overdose deaths to simulate scenarios during the COVID-19 pandemic. In this paper, we report the development of a dynamic model to simulate opioid use and opioid overdose deaths during the COVID-19.

## 2. Methods

Model development was conducted by a collaborative interdisciplinary team of mathematical modellers, epidemiologists, and a social health psychologist, and with input from those working in substance use and harms programs and policy. Model development was informed by a literature review conducted by the research team which identified and summarised the literature between January 1990 and January 2020 on dynamic models of opioid use and opioid overdose deaths. 

The model we developed is a dynamic compartmental model representing the general Canadian population aged 15 years or older, to align with the minimum age group for many sources of data on opioid use. The model involves three major sub-systems: the prescription use of opioids (short-term prescription opioid use, long-term prescription opioid use, and prescription opioid use not as prescribed), non-medical use of opioids (sometimes called illegal or illicit opioid use in other literature) and opioid overdose deaths. The model time begins in January of 2016, aligning with the start of reporting public health surveillance data for opioid overdose deaths [2] and the time scale is in months. Research Ethics Board approval was not required as this study did not collect or use any primary data from human participants. Only aggregate surveillance estimates from public facing sources were used in this model. Because only aggregate surveillance estimates were used, the investigators had no access to any potentially identifiable personal information at any time. 

**Figure 1:**
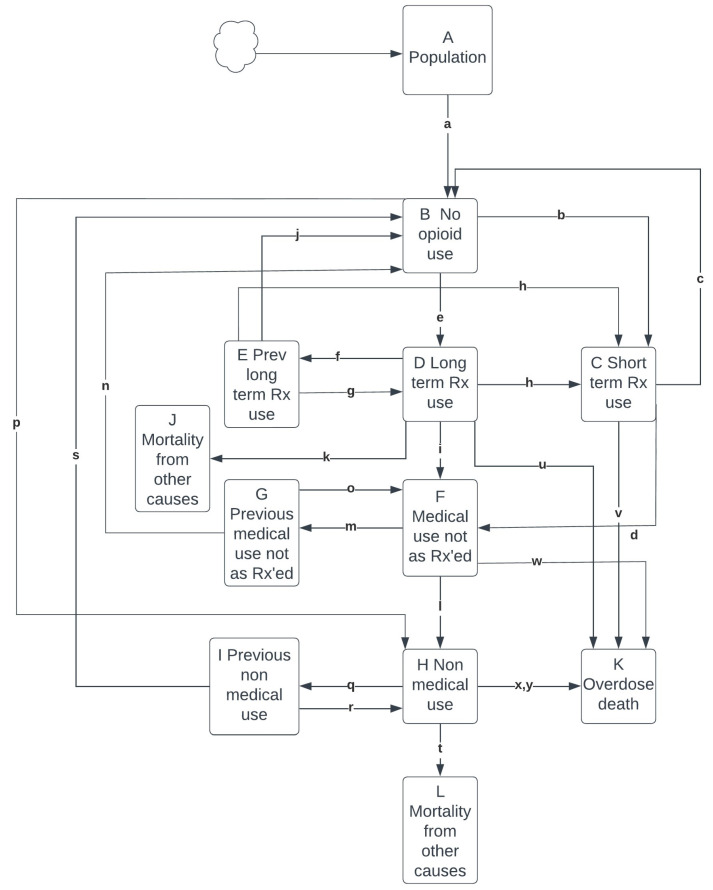
Model structure of a dynamic model of opioid overdose death. Note. Letters are used in model equations described in Table 1 and parameters described in Table 2.

Figure 1 presents a diagram of the model structure and Table 1 presents model equations. Table 2 presents model concepts, parameter names and values that appear in Figure 1 and Table 1. Tables S1 and S2 present detailed explanations about how parameter values were calculated based on evidence sources. At baseline, stocks include people who do not use opioids, those who use prescription opioids or had previous prescription use, and those who use non-medical opioids or had previous non-medical use. New people entering the population start as not using opioids and can thereafter move to non-medical opioid use or prescription opioid use. For the latter, individuals may use short-term or long-term prescription opioids, discontinue these, or move to prescription opioid use not as prescribed. Some of the population moves directly from no opioid use to non-medical opioid use, while some of those using prescription opioids not as prescribed move to using non-medical opioids. Those who discontinue opioid use may subsequently resume opioid use. Rates of resuming opioid use vary by type of opioid use. In some cases, there is a delay before moving to the no opioid use stock, during which individuals remain in a state of high risk of resumption.

People can experience opioid overdose deaths from both prescription opioid use and non-medical opioid use. Overdose mortality rates from short- and long-term prescription use, as well as prescription use not as prescribed, are assumed constant over the model period. Overdose mortality rates from non-medical opioid use are influenced by two factors: fentanyl in the drug supply and interventions implemented to prevent opioid overdose deaths (treatment and harm reduction activities); we briefly comment on each. Fentanyl is 50 and 100 times more potent than heroin and morphine, respectively and is consequentially associated with a higher rate of overdoses and deaths [31]. The level of fentanyl in the drug supply in this model is a proxy variable as we cannot observed this directly. We used the number of samples with fentanyl detected from Health Canada’s Drug Analysis Service as an indicator of fentanyl penetration and created a ratio with 2020 as the index year in order to estimate a relative measure of fentanyl in the drug supply [30]. Health interventions aimed at preventing overdose deaths are a countervailing force that reduce the rate of overdose and deaths. Since the beginning of the recognition of the opioid overdose epidemic in Canada (recognized as beginning in 2016 through the declaration of a public health emergency by the province of British Columbia and the activation of a Special Advisory Committee on the Epidemic of Opioid Overdoses by the Pan-Canadian Public Health Network of the Public Health Agency of Canada), we assume that some proportion of overdose deaths have been prevented through interventions newly implemented or increased in intensity to address the overdose epidemic. Examples of such interventions include, but are not limited to, increasing the availability of take-home naloxone, elevated access to opioid agonist therapy, growing awareness of safety measures while using opioids, and the Good Samaritan Drug Overdose Act [32,33]. 

The model input consists of the initial count of people in a particular population or stock; and the transition rates governing the flows between stocks, as shown in Table 1 and Table 2. Model inputs were identified through the peer-reviewed literature, estimates from national surveys and administrative data, and when other sources were not available, a decision by the modelling team. In the absence of published evidence on the summative effect of all measures meant to address the opioid crisis, the modelling team developed an estimate representing the proportion of deaths prevented by new health interventions implemented since January 2016.

The estimates for the proportion of deaths prevented by new health interventions to address the opioid overdose crisis implemented after January 2016 were developed through an iterative process. First, we identified the level of reduction in deaths that would be necessary for the modelled deaths to match the observed deaths, given the level of fentanyl in the drug supply. We started with a baseline value of no additional deaths prevented due to new interventions in January 2016 and assumed that new interventions implemented since then would have some cumulative effect on reducing opioid overdose deaths. We found that a value representing a reduction in deaths and increasing in a linear manner from no reduction in deaths (0% reduction) in January 2016 to a 60% reduction in December 2019, as shown in Table S3, resulted in a close visual fit of the modelled data to the observed data. This value was moderately to strongly correlated with several empirical sources of data reflecting the intensity of efforts to address the opioid crisis—take-home naloxone kit distribution in several provinces, the number of safe consumption sites operating, and level of funding provided by Health Canada through the Emergency Treatment Fund. Finally, this value also aligned with the reduction in deaths reported by a study by the British Columbia Centres for Disease Control that concluded that a combination of three interventions (safe consumption sites, take home naloxone, and opioid replacement therapy) reduced opioid overdose deaths by 60% [12]. As new surveillance data were available after 2019, values for the proportion of deaths prevented by health interventions in subsequent periods were chosen such that the simulated data approximated the observed data. It must be noted that this parameter value should not be interpreted as a casual effect; rather, it is a value developed during model development that allowed us to create a simulation where observed deaths could be approximated while taking into account an increasing level of fentanyl in the drug supply.

Following model development, parameterization, and sensitivity analysis, we calibrated the model to obtain the optimal values of three parameter values: the opioid overdose mortality rate, the fentanyl mortality factor and the prescription opioid use not as prescribed mortality factor. Within calibration, differences between the observed and simulated data were compared using the root mean squared error (RMSE) over 10,000 iterations, and the combination of parameter values from the model run with the minimized RMSE were used in subsequent simulations.

## 3. Results

**Figure 2:**
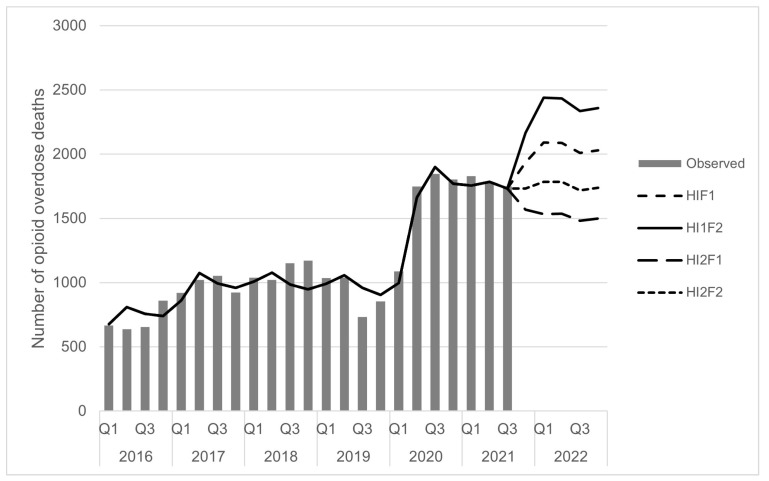
Optimized model and observed surveillance data from January 2016 to September 2021, and modelled opioid overdose deaths under four scenarios, October 2021 to December 2022. Notes: H1F1: health interventions prevent the same proportion of deaths, fentanyl remains the same; HI1F2: health interventions prevent the same proportion of deaths, fentanyl increases; HI2F2: health interventions prevent more deaths, fentanyl remains the same; HI2F2: health interventions prevent more deaths, fentanyl increases.

As shown in Figure 2, quarterly opioid overdose deaths from the calibrated model closely follow those from the empirical data for the pre-pandemic period from January 2016 to December 2019. Next, we conducted a series of simulations to account for possible changes to the opioid system during the COVID-19 pandemic using the calibrated model. These scenarios varied the proxy value describing the level of the fentanyl in the drug supply and the value used as the proportion of opioid overdose deaths prevented through health interventions. For each set of simulations, we simulated opioid overdose deaths for a total of 15 months, or five quarters. Because national surveillance data for opioid overdose deaths lag by six months and are released on a quarterly basis [2], we used the model to simulate over a period of time that had already elapsed, as well as for six months into the future. For example, in the data presented in Figure 2, which were generated in June 2022, the simulation covered the period from October of 2021 to December of 2022 [34]. Four scenarios were simulated—with either the same level of proportion of deaths prevented through health interventions or a higher level, and either the same level of fentanyl in the drug supply or a higher level. 

Simulations for the unobserved period covered a range of scenarios that varied the proportion of opioid overdose deaths prevented by health interventions and had the penetration of fentanyl in the drug supply either remain the same or increase. These assumptions were based on emerging reports of increased fentanyl in the drug supply [35], and reports that some harm reductions services may be more challenging to access, and that some harm reduction activities, like not using alone, may change in response to public health recommendations (e.g., to limiting the number of contacts and maintaining physical distance) [3,13,14]. As new data on fentanyl become available, these data were incorporated in the model. For values of fentanyl for future simulations, we used two values—either the same value as in the previous year, or an increased value that followed the general linear trend from the observed data. Figure 2 shows the simulations developed in June 2022.

**Figure 3:**
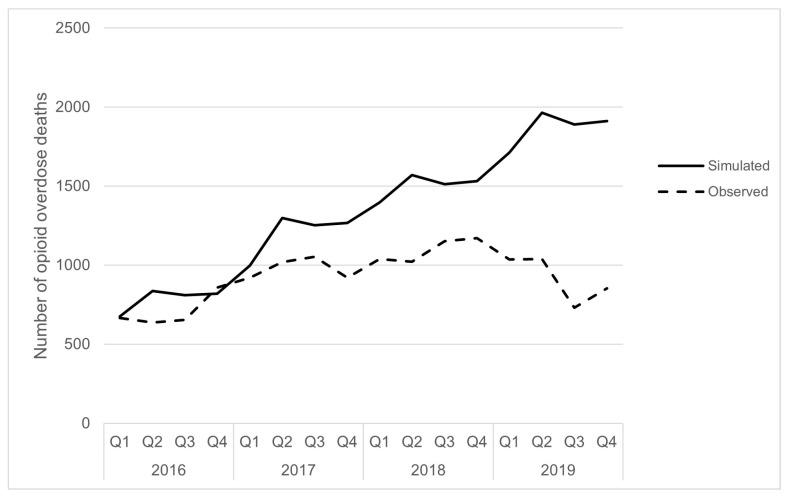
Observed surveillance data and modelled opioid overdose deaths under an alternative scenario from January 2016 to December 2019.

This model was also used develop an alternative scenario analysis, presenting one scenario of what might have happened during the first four years of the opioid crisis in the absence of new interventions, based on the assumptions in the model. For this analysis, we ran a simulation from January 2016 to December 2019 where the value of the proportion of deaths prevented through new health interventions was set to 0 for this period. Under this set of assumptions, the alternative scenario yielded a total mortality rate with an additional 6500 deaths (just under 21,500 deaths), instead of just under 15,000 deaths that were observed (Figure 3). It is critical to underscore that no causal conclusions should be made from this analysis about the effectiveness of collective efforts. Rather, this analysis can be used to demonstrate that while opioid overdose deaths increased for the first four years of the overdose crisis, under an alternative scenario, the opioid crisis could have been much worse, in the absence of new interventions implemented to reduce opioid overdose deaths, assuming that the assumptions in the model are valid. 

## 4. Discussion

Early in the COVID-19 pandemic, a concern over the concurrent opioid overdose epidemic was identified [3,13,14]. To provide one stream of evidence to inform decision-making, we developed a dynamic model of opioid overdose deaths, and simulated scenarios of opioid overdose deaths that might occur during the COVID-19 pandemic period in Canada. To our knowledge, this is the first dynamic model of opioid use and overdose death in the context of the opioid overdose crisis at the national level in Canada. Unlike in the field of infectious disease, where the susceptible-exposed-infected-recovered (SEIR) model can be quickly adapted to new and emerging infectious disease threats, there are not standard models that can be quickly adapted and applied to the opioid system to support decision making. This model, with subsequent updates, was the foundation of biannual modelling reports by the Public Health Agency of Canada.

The model presented here integrates evidence from a number of sources to provide new insights into the opioid overdose crisis, and its evolution during the COVID-19 pandemic. The opioid overdose crisis is complex and given the highly dynamic landscape of changing drug supply, changing substance use patterns, and changing interventions meant to address opioid-related harms, dynamic modelling is one tool that may help decision makers address this challenging public health concern. Importantly, this model allowed decision makers to visualize possible trajectories of the opioid overdose crisis during the concurrent COVID-19 pandemic. It also provided a platform to discuss the dynamics of the opioid overdose crisis, particularly in being able to visualize possible scenarios that could have occurred in the absence of intervention. Of the four scenarios presented in Figure 2, which were developed in June 2022, an average of approximately 1900 apparent opioid-related toxicity deaths per quarter were observed from the fourth quarter of 2021 to the end of 2022 (range of 1756–2199; [2]). This number falls within the simulated scenarios, tending towards the scenarios with a higher level of deaths. However, through the development of this model, we have identified several challenges that need to be addressed through subsequent modelling, research, and data collection efforts.

### Limitations

This model should be considered a starting point for further development. It should not be used to draw causal conclusions about the effects of interventions. Most importantly, the parameter value that is used in the model to represent the proportion of deaths that may have been prevented through health interventions was developed using the model to estimate its value and cannot be used for inference. We encourage the research community to develop and apply methods through which we can better estimate the cumulative effect of multiple interventions implemented at varying times with varying intensities within a complex system. While some studies have tried to estimate the effects of single interventions on population outcomes (such as the implementation of safe consumption sites in BC) using a natural experiment methodology [36], and others have estimated the effects of a finite set of interventions (such as take-home naloxone, opioid agonist therapy, and overdose prevention services in BC) [12], we are not aware of any studies that have tried to roll up multiple interventions aimed at addressing the opioid crisis in Canada into an index. Similar efforts during the COVID-19 pandemic have rolled up actions taken to address the COVID-19 pandemic through indices, such as the Government Response Index, Containment and Health Index, and Stringency Index [37,38]. Moreover, even the evidence on the magnitude of the effects of single interventions, such as safe consumption sites, must be interpreted in a nuanced manner, and consider the system within which the intervention exists [39]. The value for the proportion of deaths prevented by health interventions is a non-specific summary measure, the value of which is derived from the model and requires further support through future research.

While we created a monthly model, most sources of data provided quarterly or annual counts, proportions or rates. There are a number of parameters in our model that did not have readily available values, which points to the need to continue the development of relevant data sources in Canada. While the present model is able to reproduce the observed surveillance data, not all parameter values have strong empirical support. Some of our parameter values are based on data sources that exclude relevant subpopulations, such as individuals experiencing homelessness or those living in institutions, that may be more at risk for some types of opioid-related harms. This is a national model, which does not account for the known wide regional variation in how the opioid overdose crisis is unfolding is Canada [1], particularly in terms of regions hardest hit by opioid overdose deaths, and variability in the types and intensities of interventions implemented to address the crisis. The present model also does not disaggregate by age, sex, or gender, as well as additional factors that are strongly associated with opioid-related harms [2].

With these limitations in mind, we propose that this model, as a first national level, will be a useful basis for future modelling and research. This model can be extended to a longer study period and can be used to simulate future dynamics of the opioid crisis. Subsequent research and model development should explore how to effectively integrate specific interventions into the model, to better allow decision-makers to explore the possible outcomes of specific policy and program decisions, while keeping in mind the limitations of this type of modelling. Province- or region-specific, and age-, sex-, and gender- stratified models should be developed. Additional, novel sources of data such as social media data may be included in the model to provide information on the non-medical use of opioids among the Canadian population, as this has been found to be a useful source of data in other studies [40,41].

## 5. Conclusions

We developed a national level mathematical model to simulate the opioid overdose crisis during the COVID-19 pandemic. We used this model to develop simulations for what might happen to the level of opioid overdose deaths in Canada during the COVID-19 pandemic, given possible changes to the level of fentanyl in the drug supply and changes to the level of opioid overdose deaths prevented by health interventions. This model can serve as a starting point for the development of future models that can better incorporate empirical evidence on the impact of interventions.

## Figures and Tables

**Table 1:** Model equations.

No opioid use
(a) Monthly population increase = A * α
Prescription opioid use
Short-term prescription opioid use
(b) Starting short-term use = B * β * γ
(c) Stopping short-term use = C * δ
(d) Short-term use to use not as prescribed = C * ε/10
Long-term prescription opioid use
(e) Starting long-term use = B * β * (1.0 − γ)
(f) Stopping long term use = D * ζ
(g) Previous long-term use to long term use = E * η * (1.0 − γ)
(h) Previous long-term use to short-term use = E * η * γ
(i) Long-term use to use not as prescribed = D * ε
(j) Previous long-term use to no use = E * θ
(k) Long term-use all-cause mortality = D * ι
Prescription use not as prescribed
(l) Use not as prescribed to non-medical use = F * κ
(m) Stopping use not as prescribed = F * μ * 2
(n) Previous use not as prescribed to no use = G * ν
(o) Restarting use not as prescribed = G * (ξ/2)
Non-medical use
(p) Starting non-medical use = B * λ
(q) Stopping non-medical use = H * μ
(r) Restarting non-medical use = I * ξ
(s) Previous non-medical use to no use = I * ν
(t) Non-medical use all-cause mortality = H * σ
Overdose mortality
(u) Long-term use overdose mortality = D * ο
(v) Short-term use overdose mortality = C * π
(w) Use not as prescribed overdose mortality = F * ρ * χ * φ * τ
(x) Non-medical–non-fentanyl-related overdose mortality = H * ρ * φ * τ
(y) Non-medical-fentanyl-related overdose mortality = H * ρ * ψ * υ * φ * τ

**Table 2:** Concepts, parameter names, and values.

Concepts	Parameter	Value	Evidence Source
Population	A	30,874,000	[15]
No opioid use	B	A – (C + D + E + F + G + H + I)	
Short-term prescription use	C	262,170	[16]
Long-term prescription use	D	690,594	[16]
Previous long-term prescription use	E	700,000	
Prescription use not as prescribed	F	320,256	[17]
Previous prescription use not as prescribed	G	100,000	
Non-medical use (illicit and illegal opioids)	H	200,000	[18,19]
Previous non-medical use	I	No initial value provided	
Mortality from other causes among those using long-term prescription opioids	J	No initial value provided	
Mortality from opioid overdose	K	No initial value provided	
Mortality from other causes among those using non-medical opioids	L	No initial value provided	
Population increase	α	0.014 per year 0.00117 per month	[15]
Rate starting opioid prescription varies during first four years	β		[20]
Opioid Rx 2016	2016	0.0866/12 per month	
Opioid Rx 2017	2017	0.0838/12 per month	
Opioid Rx 2018	2018	0.081/12 per month	
Opioid Rx 2019	2019	0.0782/12 per month	
Proportion new short-term prescription use	γ	0.9	[20]
Rate stopping short-term prescription use	δ	0.9 per month	[20]
Rate prescription use to use not as prescribed	ε	0.093 per year 0.00775 per month	[21]
Rate stopping long-term prescription use	ζ	0.017 per month	[20]
Rate previous long-term to new prescription use	η	0.65 per year 0.55 per month	[20]
Rate to no long-term prescription use	θ	0.08796 per year 0.00733 per month	[20]
Rate of all-cause mortality among those using long-term prescription use	ι	0.06 per year 0.005 per month	[22]
Rate of moving from prescription use not as prescribed to non-medical use	κ	0.065 per year 0.0054 per month	[23]
Rate start non-medical use	λ	0.00006 per year 0.000005 per month	[24]
Rate stop using prescription not as prescribed and non-medical use	μ	0.09 per year 0.0075 per month	[25]
Delay to no use	ν	0.028	
Rate restart non-medical use	ξ	0.20 per year 0.017 per month	[26]
Rate of opioid-related mortality from long-term prescription use	ο	0.0000582769 per month	[27]
Rate of opioid-related mortality from short-term prescription use	π	0.0000043333 per month	[21]
Rate of opioid-related mortality from prescription opioid use not as prescribed and non-medical use			
Initial value	ρ	0.000542 per month	[28]
Optimized value	ρ’	0.000318 per month	
Rate of mortality from all other causes among people with non-medical use	σ	0.000783 per month	[29]
Seasonality value	τ	1.02, 1.01, 0.95, and 0.99 for Quarters 1 through 4	[2]
Proxy for level of fentanyl in the drug supply	υ	2016—0.25 2017—0.48 2018—0.61 2019—0.08 2020—1.00 2021—1.22	[30]
Level of deaths prevented through health interventions	φ	See Table S3	See Section 2
Mortality multiplication factor for prescription use not as prescribed			
Initial value	χ	1	
Optimized value	χ’	0.1	
Mortality multiplication factor for fentanyl			
Initial value	ψ	5	
Optimized value	ψ’	10	

Note: For detailed explanations of each parameter, see Tables S1 and S2.

## Data Availability

The data presented in this study are available from the Public Health Agency of Canada at: https://health-infobase.canada.ca/src/doc/HealthInfobase-SubstanceHarmsData.zip (accessed on 27 January 2023).

## Supplementary Materials

The following supporting information can be downloaded at: https://www.mdpi.com/article/10.3390/ijerph21040442/s1, Table S1. Initial values of stocks, evidence sources and detailed explanations. Table S2. Model parameter values, evidence sources and detailed explanations. Table S3. Value of level of deaths prevented through health interventions, as described in methods.
