# Supplementary material for: A Dynamic Model of Opioid Overdose Deaths in Canada during the Co-Occurring Opioid Overdose Crisis and COVID-19 Pandemic
Source: Int J Environ Res Public Health. 2024 Apr 4;21(4):442. doi: 10.3390/ijerph21040442 (PMC11050073; doi:10.3390/ijerph21040442)
Supplement: Supplementary file 1 [file ijerph-21-00442-s001.zip › ijerph-2902346-supplementary.pdf]

Table S1. Initial values of stocks, evidence sources and detailed explanations.

Concept	Stock	Initial Value	Source of Evidence
**Population**	A	3.0874E-7	Statistics Canada. Table 17-10-0005-01 Population estimates on July 1st, by age and sex, population 15+ [15]
**No opioid use**	B	A – (C + D + E + F + G + H + I)	Calculated from other starting values
**Short-term prescription use**	C	262,170	Calculated based on value from CIHI report applied to 2016 population [16]
**Long-term prescription use**	D	690,594	Calculated based on value from CIHI report applied to 2016 population [16]
**Previous long-term prescription use**	E	700,000	Modeling team judgement, based on estimate in D
**Prescription use not as prescribed**	F	320,256	Statistics Canada estimate from 2018 Canadian Community Health Survey pain relieving medication module, scaled to 2016 population [17]
**Previous prescription use not as prescribed**	G	100,000	Modeling team judgement
**Non-medical use (illicit and illegal opioids)**	H	200,000	Conservative mean estimate is 500 per 100,000 based on coroner's data; 511.8 per 100,000 in another paper; [18] [19]
**Previous non-medical use**	I	No initial value provided	
**Mortality from other causes among those using long-term prescription opioids**	J	No initial value provided	
**Mortality from opioid overdose**	K	No initial value provided	
**Mortality from other causes among those using non-medical opioids**	L	No initial value provided	

Table S2. Model parameter values, evidence sources and detailed explanations.

Concept	Parameter Name	Parameter Value	Notes	Evidence Source
Population Increase	α	0.014 per year<br>0.00117 per month	Calculated as the monthly percent population increase from 2018 - 2019	[15]
Rate starting opioid prescription varies during first four years	β	n/a	Calculated the average yearly decrease based on prevalence of 9.5% in 2013 and 8.1% in 2018. Values for 2016, 2017, and 2019 were calculated based on a 0.28% per year change	[20]
Opioid Rx 2016	2016	0.0866/12 per month		
Opioid Rx 2017	2017	0.0838/12 per month		
Opioid Rx 2018	2018	0.081/12 per month		
Opioid Rx 2019	2019	0.0782/12 per month		
Proportion new short-term prescription use	γ	0.9	Proportion of people starting opioids whose duration of use is less than 30 days	[20]
Rate stopping short-term prescription use	δ	0.9 per month	90% of people started opioids have a duration of less than 30 days	[20]
Rate prescription use to use not as prescribed	ε	0.093 per year<br>0.00775 per month	9.3% of people with a long-term prescription go on to addition	[21]
Rate stopping long-term prescription use	ζ	0.017 per month	20% of people on long-term therapy stopped for at least six months in 2017	[20]
Rate previous long-term to new prescription use	η	0.65 per year<br>0.55 per month	2/3 of those on long-term therapy who stopped for at least six months started an opioid again in the next year	[20]
Rate to no long-term prescription use	θ	0.08796 per year<br>0.00733 per month	4.4% of people stopped long-term prescription opioids and changed to a non-opioid pain medication within six months, which is used to move people from the stopping long-term prescription stock to the no opioid use stock	[20]
Rate of all-cause mortality among those using long-term prescription use	ι	0.06 per year<br>0.005 per month	6% of those with long term opioid initiation died within a year of follow up from all cause mortality	[22]

Rate of moving from prescription use not as prescribed to non-medical use	κ	0.065 per year 0.0054 per month	Fishbain (2008) article provides prescription use to addiction. This parameter is use not as prescribed to non-medical use. Half the lower bound as the parameter value 0.204 (range: 0.13 – 0.4)	[23]
Rate start non-medical use	λ	0.00006 per year 0.000005 per month	n/a	[24]
Rate stop using prescription not as prescribed and non-medical use	μ	0.09 per year 0.0075 per month	Rate of remission from opioid dependence – conservative estimate 0.0917 (95% CI 0.0842, 0.0979)	[25]
Delay to no use	ν	0.028	Represents a 3-year delay to move from previous non-medical use to no use	Modelling team judgement, based on a conservative judgement of how long it would take to return to the same risk as the general population
Rate restart non-medical use	ξ	0.20 per year 0.017 per month	Caldwell uses a value ranging from 0 to 45% per month for relapse post-rehab; this is more conservative rate	[26]
Rate of opioid-related mortality from long-term prescription use	ο	0.0000582769 per month	59 opioid related deaths among 32,449 patients on long-term opioid therapy, deaths occur at a median of 2.6 years	[27]
Rate of opioid-related mortality from short-term prescription use	π	0.0000043333 per month	5.2 per 100,000 per year	[21]
Rate of opioid-related mortality from prescription opioid use not as prescribed and non-medical use				
Initial value	ϱ	0.000542 per month	Annual crude mortality rate due to opioid overdose among people who use opioids; 0.65 per 100 people per year	[28]
Optimized value	ϱ′	0.000318 per month		
Rate of mortality from all other causes among people with non-medical use	σ	0.000783 per month	1.59 per 100 PY minus rate of mortality from non-medical opioids (0.65 per 100 PYs)	[29]

Seasonality value	τ	1.02, 1.01, 0.95, and 0.99 for Quarters 1 through 4	Calculated based on pre-pandemic quarterly variation in opioid overdose deaths [2]
Proxy for level of fentanyl in the drug supply	υ	2016 – 0.25<br>2017 – 0.48<br>2018 – 0.61<br>2019 – 0.08<br>2020 – 1.00<br>2021 – 1.22	Health Canada Drug Analysis Services [30] Ratio of annual number of samples analysed by containing fentanyl per year, 2020 as reference year
Level of deaths prevented through health interventions	φ	See Appendix 3	Value developed by modelling team as described in methods
Mortality multiplication factor for prescription use not as prescribed			
Initial value	χ	1	Modelling team judgement for starting value and range, optimized value determined through calibration, values could range from 0.1 to 1
Optimized value	χ′	0.1	
Mortality multiplication factor for fentanyl			
Initial value	ψ	5	Modelling team judgement for starting value and range, optimized value determined through calibration, values could range from 1 to 10
Optimized value	ψ′	10	

Table S3. Value of level of deaths prevented through health interventions, as described in methods.

Year	Quarter	Month	Value
		0	1.0
**2016**	1	1	1.0
	1	2	0.99
	1	3	0.97
	2	4	0.96
	2	5	0.95
	2	6	0.94
	3	7	0.92
	3	8	0.91
	3	9	0.9
	4	10	0.89
	4	11	0.87
	4	12	0.86

**2017**	1	1	0.85
	1	2	0.83
	1	3	0.82
	2	4	0.81
	2	5	0.8
	2	6	0.78
	3	7	0.77
	3	8	0.76
	3	9	0.74
	4	10	0.73
	4	11	0.72
	4	12	0.71
**2018**	1	1	0.69
	1	2	0.68
	1	3	0.67
	2	4	0.66
	2	5	0.64
	2	6	0.63
	3	7	0.62
	3	8	0.6
	3	9	0.59
	4	10	0.58
	4	11	0.57
	4	12	0.55
**2019**	1	1	0.54
	1	2	0.53
	1	3	0.51
	2	4	0.5
	2	5	0.49
	2	6	0.48
	3	7	0.46
	3	8	0.45
	3	9	0.44
	4	10	0.43
	4	11	0.41
	4	12	0.4
